# Pros and Cons of Antigen-Presenting Cell Targeted Tumor Vaccines

**DOI:** 10.1155/2015/785634

**Published:** 2015-10-25

**Authors:** Cleo Goyvaerts, Karine Breckpot

**Affiliations:** Laboratory of Molecular and Cellular Therapy, Department of Biomedical Sciences, Vrije Universiteit Brussel, 1090 Brussels, Belgium

## Abstract

In therapeutic antitumor vaccination, dendritic cells play the leading role since they decide if, how, when, and where a potent antitumor immune response will take place. Since the disentanglement of the complexity and merit of different antigen-presenting cell subtypes, antitumor immunotherapeutic research started to investigate the potential benefit of targeting these subtypes *in situ*. This review will discuss which antigen-presenting cell subtypes are at play and how they have been targeted and finally question the true meaning of targeting antitumor-based vaccines.

## 1. Introduction

Active immunotherapy aims to administer the appropriate tumor associated antigens (TAAs) in such a way that antigen-presenting cells (APCs) can process and present them to oncolytic effector cells in order to eradicate primary and metastasized cancer cells. Since the dendritic cell (DC) is the most professional APC,* ex vivo* loaded and stimulated DCs were initially used to achieve this response. However, the* ex vivo* generation and modification of DCs turned out to be a labor-intensive, time- and money-consuming procedure. Furthermore, the variability in DC sources, techniques, and vectors used for TAA transfer led to much diversity in reported TAA expression [1]. Moreover, the* in vitro* generated DCs may not represent the most suited DC subtype for the induction of a CD4^+^ T helper 1 (T_H_1) polarized antitumor immune response. To reduce the DC generation linked variability and exploit the functional characteristics of relevant DC subtypes, naturally circulating DCs have been tested for the induction of long-lasting clinical benefits [2]. Nevertheless, as the isolation of patient-specific DCs remains a labor-intensive and expensive task, direct targeting of TAAs to DCs* in situ* represents a straightforward and therefore preferred strategy. Moreover, direct delivery of cargo to DCs* in situ* could offer additional benefits such as (1) generation of scalable, stable, and standardized vaccines, (2) the ability to tune the direction and strength of the immune response (humoral versus cell-mediated), and (3) improvement of the vaccine's safety profile by reducing the required dose that ends up in nontarget cells and as such diminishing the risk on adverse events. Therefore, numerous groups have evaluated APC targeted vaccination approaches [3–8]. To remain within the scope of this review, we will limit this discussion to APC targeted strategies evaluated in the framework of antitumor immunotherapy.

## 2. Which Cells Should Be Targeted?

### 2.1. Dendritic Cells as the Most Professional Antigen-Presenting Cells

The art of antigen processing and presentation to naive T cells* via* major histocompatibility complex (MHC) classes I and II molecules is a privileged feature of three hematopoietic cell types: DCs, macrophages, and B lymphocytes. While the latter two also conduct other functions in innate and humoral immunity, respectively, the former are the most professional and fulltime APCs and are up to 1000-fold more efficient in activating resting T cells [9]. The fact that DCs are specialized APCs is reflected in numerous phenotypic and functional features.

#### 2.1.1. Phenotypically

DCs are characterized by stellate cytoplasmic protrusions, which endow them with an elongated contact surface for antigen capture and presentation [10]. Their specialized antigen capturing features are further evidenced by the notion of several antigen uptake receptors such as DC inhibitory receptor 2 (DCIR2) and DEC205 [11] next to their unique capability to cross-present exogenous antigens to CD8^+^ T cells upon uptake of draining antigens and antigen handover from migratory DCs [12] or by acquiring peptide-MHC complexes also known as “cross-dressing” [13]. As opposed to macrophages, they are further able to regulate their processing capacity and by degrading their engulfed cargo more slowly, they can control lysosomal degradation in order to preserve peptides for T-cell recognition [14]. Next to the presence of MHC/peptide complexes, DCs also express several costimulatory molecules in order to properly guide the naive T cells [15].

#### 2.1.2. Functionally

Next to these structural features, DCs have a remarkable functional plasticity. To accomplish this, they are strategically positioned at body barriers and organ entry ports [16]. On the one hand, they are able to induce immune responses against invading pathogens (nonself). On the other hand, DCs can induce tolerance in order to avoid unwanted immune reactions against autoantigens (self) [17]. In general, immature DCs efficiently take up pathogens, apoptotic cells, and particulate antigens from the environment by receptor-mediated phagocytosis, macropinocytosis, or caveolae and clathrin-mediated endocytosis. Furthermore, they remain tissue-resident, have a high turnover rate of MHC-II/peptide complexes, and lack T-cell stimulatory molecules and hence induce T-cell energy instead of T-cell activation upon DC-T cell interaction [18]. In contrast, activated DCs are considered to be immunogenic. Upon maturation, they lose their endo- and phagocytic receptors and slow down their antigen capture and processing rate, while they upregulate both “signal two” molecules like costimulatory molecules (e.g., CD80 and CD86) and “signal three” molecules (e.g., IL-12) to stimulate and polarize naive T cells, respectively. Furthermore, they acquire a higher cellular motility by upregulating the C-C chemokine receptor type 7 that enables DCs to migrate from the periphery to the T-cell areas of draining lymphoid tissues. However, the view that immature DCs induce tolerance and mature DCs induce immunity is simplified. It has been demonstrated that mature DCs can contribute to T-cell tolerance as well [19], suggesting that the maturation trigger dictates the immune functions of the DCs.

#### 2.1.3. Sensitivity

The third reason why DCs are such sophisticated APCs is reflected by the complexity of maturation signals they can detect and respond to [14]. The most important pathways known today are (1) the encounter of microbial agents that trigger surface or intracellular Toll like receptors (TLRs), C type lectin receptors (CLRs), retinoic acid-inducible gene 1 (RIG-I) or nucleotide-binding oligomerization domain (NOD) like receptors [20, 21], (2) the direct interaction with cells such as B cells, T cells, natural killer (NK) cells, natural killer T (NKT), and *γδ* T cells, (3) stimulation by cellular products like CD40 ligand (CD40L), IL-1*β*, TNF*α*, and IL-6, and (4) the products of dying cells named damage-associated molecular patterns (DAMPs) like heat shock proteins, high mobility-group box 1 proteins, and uric acid [17]. As distinct antigens are able to trigger DC maturation* via* one or more of these pathways, this combination serves as a fingerprint that triggers a specific set of receptors [22, 23]. Subsequently, complex signaling networks are activated which cooperate, integrate and finally converge in the upregulation of distinct transcription factors [24].

A final hallmark of their professionalism is represented by their differential anatomical locations, expression of different markers, distinct antigen processing capacities, and variable responses to maturation stimuli or, in other words, their subdivision in specialized subtypes, as discussed in the next section.

### 2.2. Dendritic Cells as a Heterogeneous Population of Subsets

Given the plethora of antigens, their varied routes of entry into the body, and their diverse characteristics, it is not surprising that a network of professional APCs dedicated to control T-cell immunity diversified to cope with all intruders at all phases of the immune response. About 15 years ago, researchers started to investigate the complexity and merit of the different DC subtypes. However, unraveling this complexity has been complicated in part due to the rarity of DCs in tissues (≈1% of cells), their short life span, and their lack of cross-species unifying surface markers. Therefore, the field of DC subtyping came with a lot of observations, hypotheses, and contradictions. However, with the latest ontogenic, phenotypic, and genetic data, it is currently postulated that there are two main “true” DC subtypes in both mice and men: plasmacytoid DCs (pDCs) and conventional DCs (cDCs), which are further subdivided into cDC1 and cDC2. Below we will elaborate on these subtypes as well as on two cell types that were long considered to be distinct DC subtypes, namely, Langerhans cells (LCs) and monocyte derived DCs (moDCs).

#### 2.2.1. Ontogenic Level

“True” DCs are defined by their fms-like tyrosine kinase 3 ligand- (Flt3-L-) dependent development from hematopoietic stem cells into blood residing pre-cDCs and pDCs [25]. Next, the development of cDC1 is orchestrated by IFN regulatory factor-8 (IRF8), basic leucine zipper ATF-like 3 transcription factor (BATF3), nuclear factor regulated by interleukin-3 (NFIL3), and inhibitor of DNA binding 2 (Id2) [26]. When Id2 is suppressed by E2-2, pDCs are generated [27]. To differentiate into cDC2, transcription factors v-rel avian reticuloendotheliosis viral oncogene homolog B (RelB), neurogenic locus notch homolog protein 2 (NOTCH2), recombination signal binding protein for immunoglobulin kappa J region (RBP-J), IRF2, and IRF4 are employed [28]. Although presumed for a very long time, both skin residing LCs and moDCs are not considered “true” DCs since their development is Flt3-L-independent [29]. While circulating monocytes are rapidly mobilized to differentiate into moDCs under inflammatory conditions [30], LCs seem to originate from fetal liver monocytes [31] that require colony stimulating factor 1 receptor (CSF1R) engagement* via* IL-34, which suggests that LCs are more closely related to macrophages [26].

#### 2.2.2. Phenotypic Level

Initially both murine and human cDCs were defined as CD11c^+^ MHC-II^+^ cells located in lymphoid as well as nonlymphoid tissues. Furthermore, cDCs found in lymphoid tissue like bone marrow, spleen, and lymph nodes are called resident DCs and were subdivided into CD8*α* ^+^ or CD4^+^ cDCs in mice versus CD1c (BDCA-1)^+^ or CD141 (BDCA-3)^+^ cDCs in human. In the nonlymphoid tissues like skin, lungs, and gut, DCs are called migratory since they tend to migrate from peripheral tissues to lymphoid tissue through the lymphatics. In mice, these cDCs are defined to express CD103 or CD11b while, in humans, the same surface markers as in the lymphoid tissues were observed. So while in mice cDCs express different markers in different anatomical locations, human CD141^+^ DCs and CD1c^+^ DCs are abundantly present in both lymphoid and some nonlymphoid tissues such as liver, lung, and skin. Next to the cDCs, pDCs are also broadly distributed throughout the body [32]. While mouse pDCs are Lin^−^MHC-II^+^ and specifically express CD11c, B220 (CD45R), CD317 (BST2), and SiglecH, human pDCs express IL-3R*α* (CD123), CD303 (BDCA-2), and CD304 (BDCA-4). The murine moDCs express CD11b, CD11c, MHC-II, CD64, and Fc*γ*R*ε* alongside varying levels of Ly6C. The human counterparts all express high levels of MHC-II, CD11c, CD11b, CD24, CD1a, and CD206 but lose expression of both macrophage colony stimulating factor (M-CSF) receptor and Ly6C [33]. Of note, an extra subset of human dermal DCs is represented by the CD14^+^ cells which are characterized by their expression of DC-specific ICAM3-grabbing nonintegrin (DC-SIGN), C-type lectin domain family 1 member (CLEC) 6, lectin-like oxidized LDL receptor-1 (LOX-1), and dectin-1 [34]. Finally, murine LCs characteristically express langerin (CD207), a C type lectin that is localized in LC-specific organelles called Birbeck granules. Human LCs are identified as langerin^+^, DEC205^+^, CD1a^hi^, and CD11c^lo^ (Table 1).

#### 2.2.3. Genetic Level

It became clear that murine lymphoid tissue CD8*α* ^+^ and nonlymphoid tissue CD103^+^ cDC subsets as well as the lymphoid tissue CD4^+^ and nonlymphoid tissue CD11b^+^ DC subsets combined constitute two cross-species DC lineages, respectively. Therefore, it was recently proposed to subdivide cDCs into only two main subtypes: one classical type 1 DC (cDC1) for murine CD8*α* ^+^/CD103^+^ and human CD141^+^ cDCs [35] and cDC2 for murine CD4^+^/CD11b^+^ and human CD1c^+^ cDCs (Table 1). Within the cDC1 group, chemokine receptor-1 (XCR1) is emerging as an important cross-species marker, which supports the view that the traditional DC subset markers CD8*α* and CD141 are inferior identifiers of the cDC1s and are being superseded by XCR1, CLEC9A (DNGR-1), and cell adhesion molecule 1 (CADM1). Within the cDC2 group, the most conserved markers are MHC-II^hi^ and signal-regulatory protein *α* (SIRP*α* ^+^). The only known pDC-specific conserved markers are IRF7/8 next to TLR7/9 while the conserved markers of LCs are MHC-II, E-cadherin, epithelial cell adhesion molecule (EpCAM), CD11c, and langerin (CD207). Finally, while the human and murine moDCs share the conserved markers MHC-II, CD11b, and CD11c, the human moDCs are further characterized by CD16 and the murine version by Ly6C and DC-SIGN.

#### 2.2.4. Functional Level

Both murine and human cDC1 selectively express genes involved in the balance between tolerance and cross-presentation [36, 37]. They express high levels of MHC-I processing-associated proteins. In addition they possess the dual capacity to produce large amounts of type I IFN and IL-12, making cDC1 ideal stimulators of CD8^+^ cytotoxic T cells (CTLs) [14, 38]. A recent study underlined that only the intratumoral cDC1s were able to facilitate adoptive CTL control of tumor outgrowth [39].

In contrast mouse and human cDC2 efficiently present antigens to CD4^+^ T cells, favoring their polarization into T_H_2 and T_H_17 cells. They also appear to display a capacity to cross-present antigens and secrete high levels of IL-12, suggesting a potential key role in promoting IFN*γ* release by NK cells and therefore also T_H_1 polarization [40]. This redundancy for cDC1 and cDC2 may be a way to allow “mass cross-presentation” as the human CD141^+^ cDC1 represent only a small fraction (≈2%) of all DCs, at least in blood [41].

The pDCs are best known for their ability to produce high amounts of type I IFN (IFN*α* and IFN*β*) in response to viral stimuli and as such control the progress of viral infections at various levels [42]. In their resting state, however, pDCs play an important role in the induction of tolerance owing to a low expression of MHC and costimulatory molecules compared to their cDC counterparts. However, in humans this view has been challenged by recent findings that metastatic melanoma patients receiving intranodal injections of activated and peptide loaded pDCs were very effective at inducing potent antitumor immunity [43].

MoDCs are a special type of subset since they are created according to the type of inflammation. In general, moDCs capture antigen and migrate to the draining lymphoid tissues to predominantly drive T_H_1 or T_H_17 immunity by producing IL-12 or IL-23, respectively. After infection they can also produce TNF*α* and inducible nitric oxide synthase (iNOS). Furthermore, they seem to be evolved as a crucial reservoir of APCs with a potent emergency backup role in cases of acute inflammation [30].

Finally the murine epidermal LCs are an atypical APC subset that seems specialized in the uptake and processing of antigens in the periphery for peripheral tolerance induction, especially during steady state conditions. In addition, they can produce IL-23, IL-6, and IL-1*β* during inflammation. On the contrary, human LCs have been described to induce robust proliferation of naive allogeneic CD8^+^ T cells far more efficient than the CD14^+^ DCs through the secretion of IL-15 which promotes the differentiation of granzyme B^+^/perforin^+^ CTLs. Moreover, they appear to be efficient at cross-presenting peptides [44]. In general, the role of LCs seems to be dictated by environmental cues, rather than a preimprinted behavior.

In summary every DC subset has its own functional specialties, which opened up exciting possibilities for targeted manipulation to tune the immune response by harnessing subset specific attributes. Subsequently antitumor vaccination became not only a question of proper DC activation but also of selecting the most appropriate DC subtype [34, 44].

## 3. How Can Antigen-Presenting Cell Subsets Be Targeted?

In general every active antitumor vaccine needs to comprise both a TAA and an appropriate stimulus to avoid the induction of TAA specific tolerance. In terms of vaccination modalities, we can roughly subdivide them in four groups: naked protein based, naked nucleic acid based, viral vector based, and nanoparticle based vaccines [40, 45–48]. In general, both naked protein and nucleic acid based vaccines are relatively easy to generate. However, they always need to be codelivered with an adjuvant to achieve robust antitumor immunity. On the contrary, viral vectors and nanoparticles are intrinsically immunogenic as they have a pathogen-like size and appearance. Moreover, when* in vivo* vaccination of mice with a viral vector was compared to peptide, DNA, or DC vaccination, stronger tumor specific immune responses were elicited with viral vectors [49–51]. As antitumor vaccines have been developed in numerous shapes and sizes, their extent of targeting possibilities is very diversified as well. In this section we will discuss the three main targeting approaches while a detailed overview of the performed preclinical and clinical* in vivo* APC targeting experiments in the framework of antitumor immunotherapy is summarized in Table 2.

### 3.1. Administration Based Targeting

After antigen delivery the so-called “depot-effect” tends to retain most of the antigen at the injection site. To increase vaccine uptake by APCs, the most straightforward way is represented by vaccine delivery into an APC rich site such as the tumor draining lymph node or spleen. For example, when we delivered TAA encoding lentiviral vectors (LVs) or mRNA intranodally in mice, a stronger therapeutic CD8^+^ T-cell response was induced than after subcutaneous delivery [45, 52]. Alternatively vaccines can be developed in such a way that they become prone to accumulation in lymphoid organs [53–55]. The latter is exemplified by two different studies in tumor bearing mice with nanoparticles (NPs) coupled to adjuvant alone or also a TAA. The NPs accumulated in the tumor draining lymph nodes when intradermally administered in the limb ipsilateral to the tumor or in the nontumor draining lymph node when administered in the contralateral limb. Interestingly, only when these NPs were targeted to the tumor draining lymph node, the CD4^+^ T-cell distribution within the tumor repolarized towards a T_H_1 phenotype and an increased frequency of therapeutic antigen-specific CD8^+^ T cells within the tumor was observed. Together, these data implicate that the tumor draining lymph node is an appealing vaccine target for solid tumors and can be targeted with NPs [56–58]. Of note also skin DC networks have been targeted* via* the use of polymeric dissolving microneedle arrays with nanoencapsulated antigen [59, 60].

Recently, intratumoral administration of antitumor vaccines has emerged [61]: on the one hand because numerous vaccination studies showed the induction of potent TAA-specific T-cell responses without clear therapeutic benefit [62] and on the other hand because the tumor microenvironment turns out to be a very manipulative system that is able to protect tumor cells from a cytotoxic attack and moreover help in tumor progression. Noteworthy in this process are the regulatory myeloid cells, represented by myeloid derived suppressor cells, type 2 or N2 tumor associated neutrophils, a subset of mast cells, M2 macrophages, and regulatory tumor associated DCs [63]. Although the latter two could be potent antigen presenters of the TAAs they capture in their surroundings, the tolerogenic microenvironment squeezes them into a suppressive state. Interestingly, since they retain a highly plastic phenotype, it seems possible to reprogram them towards potent antitumor immunity stimulating APCs. Therefore, our understanding is shifting emphasis from targeting APCs within the draining lymphoid organs by intranodal injection or lymph node targeted NPs to reprogramming APCs within the tumor microenvironment by intratumoral injection [64].

While administration based targeting is a very straightforward way to increase the chance that APCs are stimulated, it does not allow APC subtype specific targeting. Therefore, other targeting approaches have been developed as discussed in the following paragraphs.

### 3.2. Expression Based Targeting

When a nucleic acid based vaccine is administered, the expression of the encoded TAAs is most often driven by a strong constitutive promoter with or without enhancer sequences. These include the cytomegalovirus, spleen focus forming virus, human polypeptide chain elongation factor-1*α*, phosphoglycerate kinase, and ubiquitin C promoters [65–67]. Although these promoters induce strong and ubiquitous expression of the transgene, they are (1) more prone to promoter inactivation than cell specific promoters, (2) more potent in activating the host-cell defense machinery, and (3) increasing the potential risk of insertional mutagenesis caused by their enhancer sequences [68, 69]. These downsides resulted in the development of various strategies to allow APC-specific transgene expression by incorporating cell type specific regulatory elements and/or promoter(s) in the expression cassette [70, 71]. Examples are the CD11c, DC-SIGN, DC-STAMP, langerin, HLA-DR, MHC-II, and dectin-2 promoter [72–75]. However, DC specific transgene expression does not guarantee a strong CTL response, since DC specific promoters have also been applied to induce transgene specific tolerance [76, 77]. Furthermore, it has been described that tissue specific promoters may still be active in many different cell types or states since the promoter is used outside its normal genomic context [78]. Moreover, transcriptional targeting does not reduce the possible risk for insertional mutagenesis nor the possibility of cargo transfer to germ line cells [79]. Due to these conflicting outcomes, more research needs to be done on the immune stimulatory potential of APC specific promoter driven antitumor vaccines.

### 3.3. Cell Entry Based Targeting

Cell entry based targeting exploits APC specific surface receptors to target a particular APC subtype and mediate vaccine internalization [80]. Robustly, five main APC specific receptor families have been evaluated for targeting: the CLR family, integrins, Fc*γ* receptors, MHC-II molecules, and immune stimulating receptors. Of these, CLRs have been the focus of most APC targeted research in mice, nonhuman primates, and humans (Table 2) [81, 82]. Typically CLRs recognize carbohydrate structures in a calcium-dependent manner and are as such involved in the recognition and internalization of many glycosylated self-antigens and pathogens. Subsequently, CLRs can facilitate antigen uptake, processing, antigen routing, and MHC-I and -II loading. Furthermore, we depict in Table 2 all non-CLR, noncostimulatory molecular targets, as well as costimulatory molecules, used to restrict antigen delivery to APCs on the one hand and license the APC on the other hand. In general, studies regarding the most suited receptor for the induction of potent antitumor immunity remain thus far very contradictory. For example, when mice were immunized with liposomes coupled to single chain Ab fragments (scFv) against CD11c or DEC205, the latter performed twice as good [83]. In contrast, it has also been shown that CD11c targeting was better than targeting CD205, MHC-II, CD11a, CD11b, DCIR2, or CD40 in terms of cellular and humoral immunity [40].

Because receptor ligation influences intracellular vaccine routing, receptor selection has important functional consequences concerning antigen presentation and T-cell stimulation [84]. Consequently, it seems a matter of not only targeting the most suitable DC subtype, but also targeting the most appropriate DC specific receptor to induce a tailor-made response [85]. More recently, three receptors unique for the cDC1 subset have been identified, namely, DNGR1 or CLEC9A, CADM1^+^, and XCR-1. Interestingly, they are all conserved molecules across different species that are also mechanistically involved in the antigen cross-presentation process [86]. Where CLEC9A is involved in the uptake of antigen derived from apoptotic/necrotic cells, CADM1 binds to CD8^+^ T cells and mediates DC:CD8^+^ T-cell adhesion, while XCR1 promotes the functional interaction of cDC1 with NK cells and CD8^+^ T cells that secrete XCL1 [87, 88]. Since the cross-presenting cDC1 subset is currently seen as one of the most suitable targets for antitumor immunotherapy, targeting vaccines towards one of these receptors holds great promise for antitumor vaccination [39, 88].

Besides the considerable diversity in APC specific receptors, also in the approach to target these receptors there appears to be plenty of choice.

Based on the overview in Table 2, we can conclude that most studies target their vaccine by coupling it to a short peptide, a ligand, a mAb, or carbohydrate. While the latter has been used extensively to target DCs* in situ*, they mainly rely on CLR binding, which results in APC but not DC subtype specific binding. On the contrary, the former three moieties could be generated to bind one particular APC subset specific receptor. Other advantages of short peptides are that they do not severely disrupt the original vaccine formation and that targeted peptides with strong binding affinity and unlimited specificity could be generated* via* high-throughput library approaches [89, 90]. However, they can hinder multimerisation of monomers, create fusion products with lower thermostability, and hinder proper intracellular trafficking of the vaccine [91]. Alternatively, different kinds of ligands such as cytokines and growth factors have been used [92, 93]. In addition to peptides and ligands, also mAbs and their derivatives have been evaluated for APC specific targeting. In general, scFvs offer higher specificity than short peptides but as they are larger in size, the chance that they disrupt the process of conformational changes to mediate membrane fusion increases. Therefore, scFvs are most often linked to a spacer peptide or protease cleavable peptide that permits proper conformation of both the scFv domain and targetable vaccine [94].

Recently described alternatives to the above-mentioned targeting moieties are designed ankyrin repeat proteins (DARPins) and nucleic acid based aptamers as they can be selected to become high-affinity binders to any kind of target molecule [95, 96]. Another interesting alternative lies in the antigen binding part of heavy-chain-only Abs which are found in members of the family of Camelidae [97, 98]. These antigen-binding parts are only composed of one single variable region, termed VHH or nanobody. These nanobodies have unique characteristics and offer many advantages over scFvs such as (1) high solubility, (2) ability to refold after denaturation whilst retaining their binding capacity, (3) cloning and selection of antigen specific nanobodies obviating the need for construction and screening large libraries, (4) nonimmunogenic, and (5) being fused to other proteins [99, 100]. Therefore, it is not surprising that several studies have reported on the generation and subsequent use of nanobodies for APC targeting. Examples hereof are the development of nanobodies targeting CD206 to enabling selective targeting of the MMR^hi^ M2 macrophage subset within solid tumors [101]. Furthermore, we also demonstrated that several nanobodies with yet unidentified target antigens allowed targeting of specific human and murine APC subsets, including DCs and macrophages or selective targeting of cDCs [102].

In the case of viral vectors, several additional strategies have been evaluated to alter the broad infection profile of the viral outer membrane embedded glycoproteins towards an APC specific tropism. A first strategy is represented by rational point and domain mutations of the viral glycoprotein. This is exemplified by the DC-SIGN-specific targeting strategy that is based on the fact that the Sindbis virus envelope glycoprotein consists of a fusogenic E1 protein and a cell binding E2 protein. E2 normally binds to the DC-SIGN receptor, next to the canonical viral receptor heparin sulphate, expressed by many cell types. Since both protein binding sites are physically separated, selective mutation at the E2 monomer is possible, abrogating the heparin sulfate binding part while leaving the DC-SIGN binding part intact. By pseudotyping a LV with this mutated Sindbis virus derived envelope glycoprotein, targeted infection of murine DCs after direct subcutaneous administration was achieved. Moreover, this elicited strong and therapeutic antigen specific immune responses [5, 103–105]. Besides genetic alterations, the viral surface can also be chemically engineered to alter the binding specificity. Advantages are the flexibility, speed, and controllable modification conditions [106–108]. Unfortunately, the effectiveness of the chemically modified particles strongly depended on the reaction conditions of the applied modifications [109, 110]. A final strategy to generate APC targeted LVs is based on the fact that binding and fusion functions of LVs can be separated over two distinct glycoproteins. Recently we exploited this concept to develop DC subtype specific LVs by pseudotyping them with a fusogenic but binding defective glycoprotein on the one hand and an APC specific transmembranary nanobody on the other hand. Briefly we demonstrated cDC or also pDC and macrophage specific transduction of human subsets* ex vivo* and murine subsets* in vivo* [4, 111]. Importantly, similar to the report of Ciré et al. [112], who used a MHC-II targeted approach, we showed that intranodal administration of DC targeted LVs enhanced CD4^+^ T-cell proliferation, without functional nor therapeutic benefit compared to untargeted LVs [45]. Of note, besides viral vectors also bacterial derived vectors or enzymes have been successfully used to target TAAs to DCs with subsequent maturation and induction of strong antitumor immunity [113–115].

In summary an enormous amount of studies have been performed to evidence the added value of DC targeting for antitumor vaccination. However, most receptors (DEC205, DEC206, DC-SIGN, DCIR2, LOX-1, CD11c, CD11b, Fc*γ* receptors, and MHC-II molecules) are not truly specific for one particular APC subset. Subsequently they have been described to internalize antigen by different DC subsets, different APC subsets, and even other non-APCs such as endothelial cells and thymic epithelial cells [8, 116], which hampers the evaluation of the DC subtype specific impact on the induced immune response. In addition, several conflicting reports were made in mouse versus human related studies using homologous targeting moieties [117]. For example, when MUC1 was targeted to CD206, a robust CTL response was induced in mice while a robust humoral but only moderate T-cell response was observed in adenocarcinoma patients [106]. In addition, it is difficult to compare the different APC targeting studies since they were performed with very different vaccine moieties with different sizes and surface charge, different doses, diversified formulations, and targeting approaches which can result in completely different pharmacokinetic and immunological outcomes [47]. Therefore, there is an urgent need to define the true meaning of DC subset specific targeting to serve our understanding of potent active vaccines for antitumor immunity [118].

## 4. Is Targeting a Step Forward in Vaccine Development?

Although numerous studies evaluated and confirmed the efficacy of DC subtype specific targeting for immunotherapeutic purposes, other studies question its improvement compared to untargeted delivery [40, 45, 119]. Therefore, we want to elaborate in this section on the true meaning of DC (subtype) specific targeting for active antitumor vaccination.

First of all, it has been questioned if it is really possible to target APCs “actively” as they are already specialized in the uptake of whatever antigen they encounter. When Kreutz et al. injected an anti-DEC205 Ab-antigen-adjuvant conjugate in the footpad of mice, preferential uptake by APCs was mediated by the exposed antigen derived peptide and its CpG nucleic acids rather than by the APC-specific Ab [120]. Also when NPs were decorated with mannosylated alginate or different DC specific targeting Abs, this decoration was less influential on murine DC specific particle uptake, respectively [121, 122]. Furthermore, similar observations were made by our own group with “naked” mRNA where it was shown that after its intranodal delivery mainly CD8*α* ^+^ DCs were involved in its uptake [52]. This form of “passive” APC targeting was further evidenced by our own observation that noninfectious LVs were able to induce a similar therapeutic benefit in the E.G7-OVA tumor model as the APC targeted LVs after their intranodal delivery [45]. The latter was explained by the uptake of protein contaminants present within the noninfectious LV preparations, which were presumably taken up by the APCs in a nontargeted fashion. Notably, we did demonstrate that our APC targeted LVs outperformed the noninfectious LVs in terms of CD4^+^ and CD8^+^ T-cell stimulation, suggesting that the “active” APC targeting factor does account for better DC activation than “passive” uptake of noninfectious LVs. In contrast, when DC receptor internalization parameters were investigated as well as their impact on antigen presentation outcomes, targeting did turn out to be responsible for antigen presentation after Ab targeted vaccination* in vivo*. By analyzing endocytosis of DEC205, CLEC9A, CD11c, CD11b, and CD40* in vitro*, they showed that neither the receptor expression level, speed of receptor internalization, and proportion of surface turnover nor the antigen load had an impact on MHC-I or -II mediated antigen presentation. On the contrary, CD8^+^ or CD8^−^ DC targeting did enhance MHC-II or -I mediated antigen presentation, respectively. Therefore, they concluded that receptor expression levels, speed of internalization, and/or the amount of antigen delivered could be excluded as major determinants of antigen presentation efficiency in the setting of Ab targeted vaccination [123]. One elegant approach is where receptors are upregulated prior to their targeting. This was investigated with a CD206 targeting cancer vaccine composed of mAb fused to an oncofetal protein. They showed that humoral responses to low vaccine doses could be enhanced by prior administration of GM-CSF, which upregulated CD206 expression in human mannose receptor transgenic mice, while coadministration of TLR agonists was required to elicit T_H_1 immunity [124]. However, by prior administration of GM-CSF, one could question if it is the CD206 expression or the overall amount of APCs that is enhanced* in situ*. From these studies we can conclude that active APC targeting is still debatable and that more studies are warranted to unravel this enigma.

From a practical point of view it has been hypothesized that APC targeted delivery of vaccines could reduce their dose requirement. Hereby one has to distinguish two concepts: (1) APC specific targeting that results in an increased uptake of the vaccine in the case of otherwise “naked” molecules and (2) APC specific targeting to detarget vaccine delivery from all non-APCs in the case of infectious agents such as viral vectors. When APC specific uptake is enhanced, this generally results in a drastically reduced dose requirement [125, 126]. On the contrary, when we evaluated the CTL inducing capacity of broad tropism versus DC targeted LVs encoding OVA, we could not demonstrate a substantial benefit of DC targeted LVs over broad tropism LVs in terms of dose requirement without loss of efficacy [45]. Another striking observation was made by comparing different doses of* in situ* delivered targeted versus untargeted infectious nonreplicative OVA-encoding adenoviral vectors. While targeted delivery outperformed untargeted delivery after low dose administration, more effector CD8^+^ T cells were induced with high doses of untargeted vaccine compared to targeted delivery. Interestingly, the protective capacity of the nontargeted vaccine was superior to that of the targeted vaccine in a tumor challenge model, demonstrating dose-dependent effects of DC targeting on the quality of the induced immune response [127].

In terms of safety, vaccine detargeting from nonimmunogenic stromal cells could reduce the risk of adverse reactions such as the development of autoimmunity and the induction of tolerance or unwanted systemic cytokine release due to overstimulation [119, 128, 129]. Indeed, targeted delivery of TLR agonists reduced their dose requirement by 100-fold and was associated with a decreased serum cytokine storm and related toxicities* in vivo*, compared to administration of soluble adjuvants [130]. Furthermore, APC targeting potentially reduces the risk for insertional mutagenesis when DNA and LV-based vaccines are directly administered since APCs are differentiated short-living cells which are unlikely to transform into malignant cells.

Next, APC subtype specific targeting is believed to allow the induction of a very specific fine-tuned immune response. Since the disentanglement of the heterogeneity of different APC subtypes, their specific targeting paved the way towards fundamental research on the exact therapeutic role of each APC subset in antitumor immunotherapy. Moreover, DC subtype specific targeting has already been reported for CLEC9A/BDCA-3 (murine/human cDC1), DCIR-2 (murine cDC2), BDCA-2 (human pDCs), SiglecH and BST2 (murine pDCs), and langerin (LCs) and showed promising differences in the elicited immune responses as a reflection of the specific function of these DC subtypes* in situ* [11, 81, 131–135]. However, DC subset specific functions are not fixed but vary among several factors such as species and inflammatory state. This is exemplified by the presumed most favorable target for antitumor vaccination: the most professionalized cross-presenting XCR1/CLEC9A^+^ cDC1 subset [88]. While they are peculiarly equipped to cross-present antigens from dead cells, they seem equally potent to cross-present soluble antigens when compared to other DC subtypes [136]. Of note, in mice the cDC1 subset represents the main IL-12 producing population, while, in humans, IL-12 production is not limited to the CD141^+^ subset. Importantly, the lymphoid tissue CD8*α* ^+^ and nonlymphoid tissue CD103^+^ DCs are also mediators of systemic and intestinal tolerance, respectively. Thus, the cDC1 lineage responds to its local microenvironment in order to induce either tolerance or cross-presentation dependent CD8^+^ T-cell immunity. In line with these observations, the human BDCA3^+^ DC equivalents of the murine DC8*α* ^+^ cDCs have also been shown to excellently cross-present antigens on the one hand but to suppress an immune response on the other hand by secreting IL-10 and inducing Tregs [137]. Nevertheless, when CLEC9A and XCR1, specific for cDC1, were targeted, this approach appeared potent to eradicate established melanomas [88, 138]. Interestingly, however, it was also shown that when CLEC9A was coupled to polyI:C, curdlan or nothing, the vaccine was able to modulate CD4^+^ T cells into T_H_1, T_H_17 or Tregs, respectively [139], suggesting that the embedded adjuvant in the vaccine is more decisive for the immunological outcome than the cell type specific receptor towards which it is targeted.

Finally, targeting is believed to enhance the vaccine's immune stimulatory potential since detargeting TAAs from non-APCs but also B cells and macrophages could avoid the induction of tolerance or rapid antigen degradation. Indeed, when mice were treated with OVA, coupled or fused to Abs against DEC205, a more than 100-fold efficient and potent response was measured compared to untargeted antigens [128, 131]. Furthermore, several preclinical and clinical trials have demonstrated the effectiveness of APC targeted vaccines for human immunotherapy, which are summarized elsewhere [140]. However, most studies evaluating targeted antitumor vaccination are based on targeting receptors such as DEC205 and the mannose receptor CD206 (see Table 2), which are not specifically expressed by one particular DC subset. Therefore, it is hard to draw any conclusions with regard to an enhanced efficiency for antitumor immunity upon exclusive targeting of one DC subset. Moreover, when we targeted cDCs alone or also pDCs and macrophages using nanobody displaying LVs encoding OVA, we only observed clear differences in the induced CD4^+^ T-cell profiles, while the therapeutic outcome of the cDC and the cDC as well as pDC targeted vaccine was comparable but most importantly less strong than that of the broad tropism LV vaccine [45]. Furthermore, it has been questioned if targeting as such is responsible for the increased immunogenicity compared to untargeted delivery, since this increase has also been ascribed to the immunomodulating role of the targeting moiety itself and less by targeting the specific DC subset [120, 121, 141].

So, based on our current knowledge, there is no strong rationale to target one DC subset over another to prime TAA specific CTLs and additional* in vivo* studies with human DC subset specific targets are definitely needed to identify the most specialized DC subsets, if any [142], a rationale that is further signified by reports on bystander maturation of cDCs by pDCs as well as on the need for multiple DC subset activation for optimal T_H_1 and effector T-cell immunity [6, 14, 143]. This is exemplified by a study where the combination of BDCA3 and DC-SIGN targeted NPs was superior to targeting either subset alone in terms of T-cell activation. The mechanism underlying the observed synergy involved IL-15-dependent DC-DC cross talk suggesting that targeting only one APC subset could deprive the resultant immune response from the benefit of cross talk between different DC subsets [144]. Therefore, upcoming treatment paradigms should aim to include several primary DC subsets in a single vaccine as preclinical studies identified synergistic effects between various APCs [145].

## 5. Conclusions

An overload of targeted vaccination studies demonstrate that vaccination can be tailor-made to induce a particular phenotype of adaptive immunity by specifically targeting different surface molecules on DC subsets [146]. Nonetheless, conflicting results regarding the outcome of targeted vaccines to induce therapeutic antitumor immunity also stress that the benefit of targeting as such may not be overestimated. The immunogenicity of every vaccine, irrespective of its targeting abilities, is also characterized by its dose, size, surface charge, cargo, presence of adjuvants, route of administration, and the species to which it is delivered. So the question remains: does one targetable and omnipotent DC subtype really exists to increase the efficiency of current antitumor vaccination strategies?

## Figures and Tables

**Table 1:** Concise overview of the ontogenic, phenotypic, and functional features of the five main DC subtypes: cDC1, cDC2, pDC, LC, and moDC.

	cDC1	cDC2	pDC	LC	moDC
Ontogeny	HSC + Flt3-L, BATF3, NFIL3, and Id2	HSC + Flt3-L, RelB, NOTCH2, RBP-J, IRF2, and IRF4	HSC + Flt3-L and E2-2	Blood residing monocytes + inflammation	Fetal liver monocytes + CSF1R

Mouse Other markers	CD8*α* ^+^/CD103^+^ cDC DEC205^+^	CD4^+^ CD11b^+^ cDC	SiglecH^+^ BST2^+^ pDC B220^+^	Langerin^+^ LC	CD11b^+^ moDCs CD64, Fc*γ*R*ε*, and Ly6c

Human Other markers	CD141^+^cDC CD162^hi^ DEC205^hi^	CD1c^+^cDC CD11b^lo/+^	CD123^+^ pDC BDCA-2^+^, BDCA-4^+^	Langerin^+^ LC DEC205, CD1a^hi^	CD11b^+^ CD1a^+^ moDCs CD24^+^, CD206^+^, CD16^+^, and DC-SIGN

Conserved (besides CD11c and MHC class II)	TLR3^+^ CADM1^+^ XCR1^+^ CLEC9A^+^	MHCII^hi^ SIRP*α* ^+^	TLR7^hi^ TLR9^hi^	E-cadherin^+^, EpCAM^+^, and langerin^+^	CD11b^+^

Functions	T_H_1 Cross-presentation	T_H_2 and T_H_17 Cross-presentation	IFN-*α*/*β* and IFN*λ* Humoral	Adaptable MOUSE: Treg or T_H_17 HUMAN: IL-15 promoting CTLs + Cross-presentation	Highly adaptable (IL-12, IL-23, TNF*α*, and iNOS)

**Table 2:** Summary of *in vivo* APC targeting studies in the framework of antitumor vaccination.

Targeting moiety	Injection	Content	Effect	References
CLR				

*DEC205* *α*-GalCer NP	fp	OVA	↑iNKT, ↓growth in B16F10, and EG7-OVA (P + T)	[147]
Selected nucleic acid aptamer	i.v.	OVA	↑CD8, ↓growth OVA-B16 tumor (T if OT-I transfer)	[95]
Anti-CD11c and DEC205 scFv coupled to NP	i.v.	OVA + ADJ	↑CD8, ↓growth OVA-B16 tumor (P)	[83]
mAb fused protein	s.c.	OVA + ADJ	↑CD8, ↓growth OVA-B16 (P + T)	[131]
mAb fused protein	i.p.	HER2 + ADJ	↑CD8, ↑CD4, ↑humoral, and ↓growth neu-expressing mammary tumor (P)	[148, 149]
mAb fused protein	i.p.	Mesothelin + ADJ	↑cross-presentation, ↑CD4, ↑humoral, and ↓growth neu-expressing mammary tumor (P)	[150]
scFV modified adenoviral vector	fp	OVA	↑T cell, ↑humoral (at low doses), ↓growth OVA-B16 (P) BUT better for untargeted vectors	[127]
mAb fused protein	fp	OVA + ADJ	↑CD8, ↓growth B16 pseudo-metastasis model (P + T)	[120]
Bacteriophage displaying scFV	fp	OVA	↓growth B16F10 (Pro + Ther)	[151]
mAb fused protein	s.c.	Trp2 and gp100 + ADJ	↑CD8, ↑CD4, and ↓growth B16 melanoma (P + T)	[152]
**scFV fused to DNA vaccine**	**i.m.**	**Her2/neu + CPM**	**↑CD8, ↑humoral, long lasting memory ↓growth HER2/neu** ^**+**^ ** D2F2/E2 breast tumor + spontaneous mammary carcinomas (P + T)**	[153]
**Phase I clinical trial with CDX-1401 = human mAb fused protein **	**i.d.**	**NY-ESO-1 + ADJ**	**Patients with advanced malignancies: ↑cellular, ↑humoral (T)**	[154]

*DEC206* Mannosylated NP	s.c.	OVA + ADJ	↑T_H_1 cell, ↑humoral, and ↓growth B16F10 (P + T)	[155]
mAb fused to protein	s.c.	OVA + ADJ	↑T cell, ↑humoral, and ↓growth B11-OVA (P)	[156]
Mannan coupled protein	i.p.	MUC-1	↑CD8, ↓growth P815 mastocytoma (T)	[157]
Mannose coupled dendrimer	i.d.	OVA	↑CD8, ↑CD4, ↑humoral, ↓growth B16-OVA (P)	[158]
Mannosylated NP	s.c.	ErbB2/HER3 + ADJ	↓growth huErbB2^+^ renal carcinoma cells (T)	[125]
Mannan coated liposome-protamine-DNA	U	HPV16 E7	↓growth E7^+^ TC-1 (P + T)	[159]
Mannosylated and/or histidylated NP loaded with mRNA	i.v.	MART-1	↑CD8, ↓growth B16F10 (P)	[160, 161]
Mannan or pullulan NP complexed with protein	U	HER2	↑CD8, ↓growth HER2^+^ tumors (P + T)	[162]
D-mannose conjugated lipid-core peptide system	s.c.	HPV16 E7	↓growth TC-1 HPV-16 tumor (P)	[163]
**Clinical trial with mannan coupled protein**	**s.c.**	**MUC-1**	**↑humoral, less ↑CD8, protection against recurrence in breast cancer patients**	[106]
**Two phase I studies with CDX-1307 = hCG-*β* fused to mAb**	**i.d. or i.v.**	**hCG-*β* + ADJ **	**↑humoral and T cell with clinical benefit in patients with advanced epithelial malignancies**	[164]
*DC-SIGN* LV pseudotyped with point-mutated Sindbis virus glycoprotein	i.d.	OVA or PSCA	↑CD8, ↑CD4, and ↓growth transgenic adenocarcinoma, E.G7-OVA and PSCA-expressing B16-F10 (P + T)	[5, 103, 165]
mAb coupled protein	U	KLH	↓growth human Burkitt's lymphoma cell line in humanized mice (P)	[166]
IDLV pseudotyped with engineered Sindbis virus glycoprotein **+ currently tested in Phase I clinical trial ID-VP02**	s.c.	NY-ESO + Vpx	↑CD8, ↓growth CT26 colon carcinoma cells (P + T)	[167, 168]

*LOX-1* HSP70 fused to protein	s.c.	OVA	↑CD8 and cross-priming, ↓growth E.G7 cells (P)	[169]

*CLEC9A* mAb coupled to peptide	s.c.	MUC-1 + ADJ	↑T_H_1, ↓growth MUC-1-A2K/b^+^ MC38 (P + T)	[170]
mAb coupled to peptide	s.c.	OVA + ADJ	↑CD8, ↓growth B16 lung pseudo metastases (P + T)	[138]

*DCIR2* Anti-DCIR2 or anti-DEC205 mAb coupled protein	i.p.	OVA + ADJ	↑CD8, ↑CD4 (mixed T_H_1/T_H_2), ↑humoral, and ↓growth B16F10-OVA (P + T)	[171]

Integrin				

*CD11c* Targeted lipopeptide	i.d.	OVA, WT1, tumor lysate + ADJ	↓growth for OVA: E.G7-OVA, for mWT1: mWT1–1498 cells and for tumor lysate: MHC-I^−^ B16D8 melanoma (T)	[172]
Tumor-derived plasma membrane vesicles engrafted with two CD11c binding peptides	i.v.	OVA	↑CD8, ↑humoral, and ↓growth of metastatic B16-OVA (T)	[173]

*CD11b* Adenylate cyclase- (CyaA-) based vector	i.p. vs. i.v. or i.d.	OVA vs. HPV E7	↓growth OVA-B16 or E.G7-OVA versus TC-1 (P + T)	[113, 174]
**Phase II study with ProCervix = CyaA-based vector**	**s.c. **	**HPV16 and 18 E7 + ADJ**	**Clinical phase I trial indicated good safety and local tolerance at the highest dose, ↑T + ↑viral clearance + controlled HPV recurrence**	NCT01957878

Fc*γ* receptor				

IgG1-Fc tumor cells	s.c.	TAAs	↓growth E.G7 (P + T)	[175]
HER2-Fc cDNA	i.m. + EP	HER2	Mu: ↑T, ↓growth HER2^+^ D2F2/E2 cells (P) **Hu: *in vitro* cross-processing and ↑CD8** ^**+**^ ** T cells from breast cancer patients **	[176]

MHC-II molecule				

DNA loaded dendrimer with targeting peptide	s.c.	Trp2 or gp70 vs. OVA	↑CD8, ↑humoral, ↓growth, strong for B16OVA, and weak for gp70 BUT better with EP (P) *↔* B16 with Trp2 (T)	[177]
LV pseudotyped with scFv coupled to H protein of measles virus envelope	i.v.	OVA or male HY gene	↑CD4, ↑cytotoxic, and memory CD8 BUT not to the same extent as broad tropism LVs	[112, 178]
LV pseudotyped with scFV coupled to murine leukemia virus envelope	s.c.	OVA	↑CD8 mediated IFN*γ* secretion	[179]
DNA encoding anti-MHC II and anti-CD40 scFv or chemokines (MIP-1*α*, RANTES) with scFV of idiotype	i.m. or i.d. + EP	Idiotypes	↑CD8, ↑humoral, and ↓growth Id^+^ tumors (P)	[180, 181]

Non-CLR surface marker				

*BST2* Protein fused anti-BST2 Ab	i.p.	OVA or pHEL + ADJ	↑CD4, ↑CD8, ↑humoral + ↓growth B16-OVA (P)	[182]

Undefined				

NP with cholesteryl pullulan towards medullary macrophages	s.c.	MAGE-A4 or mERK2 + ADJ	↑cross-presentation, ↓growth MAGE-A4^+^ CMS5^+^ CT26, and mERK2^+^ sarcoma cell line (P + T)	[183]
Listeria monocytogenes expressing TAAs	i.p.	VEGFR2 + ADJ	↑CD8 with epitope spreading, ↓growth breast tumors (P + T)	[114, 184]
Coronavirus vector	i.v.	MelanA or Gp33 + ADJ	↑CD8, expanded epitope repertoire, growth MelanA^+^ or gp33^+^ B16F10 (P + T)	[185, 186]
APC specific nanobody displaying LV	i.n.	OVA	↑CD4, ↑CD8, ↓growth (T) BUT not to the same extent as broad tropism LVs	[45]
ISCOM vaccine	s.c.	OVA	↑CD8, ↓growth EG-7-, B16-, or Panc-OVA (P)	[187]

Costimulatory molecule				

*CD40* PLGA-NP coated with mAb	s.c.	OVA and E7 + ADJ	↑CD8, ↑CD4 + ↓growth B16-OVA (P + T)	[188]
CD40 targeted adenoviral vector	i.p.	PSMA + ADJ	↑CD8, ↓growth RM-1-PSMA model (T)	[189]
CD40L extracellular domain to adenoviral vector in mice + **Clinical trial**	i.d.	Mice: Trp2 or gp100 **Human: MART-1**	Mice: ↑CD8, ↓growth B16F10 (T) **↑CD8 in melanoma-draining sentinel lymph nodes **	[190, 191]

*B7* Syngeneic epithelial cells continuously secreting CTLA-4-ErbB2 fusion vaccine	s.c.	HuErbB2 + IL-15	↑CD8, ↑humoral, ↓growth ErbB2^+^ renal cell carcinoma (T)	[192]

*Treml4, Ig superfamily member* mAb against Treml4	i.p.	OVA or HER2 + ADJ	↑CD8, ↑CD4, ↓growth neu^+^ mammary tumor cell line NT2.5 (P)	[193]

*TLRs* TLR9 targeting protein (via DNA sequence)	i.d.	OVA + CpG	T_H_ - independent ↑CD8 + ↓growth E.G7-OVA (P + T)	[194]
TLR2 targeting lipid moiety + epitopes	s.c.	OVA	↑CD8, ↑humoral + ↓growth B16-OVA, and Lewis lung-OVA (P + T)	[195]
TLR5 targeted peptides (via flagellin) engrafted onto liposomes	i.v.	OVA	↑maturation of DCs, ↑CD8, ↑humoral, ↓growth B16, and P815 (P + T)	[173, 196]
TLR4 targeting protein (via fibronectin)	i.t. or i.v.	HPV E7 w or w/o ADJ or CPM	↑CD8 with cure of established TC-1 tumors i.t.: in the absence of additional ADJ i.v.: when + ADJ or CPM + ADJ	[197]
TLR4 targeting protein (via fibronectin) + anti-CD40, TLR3 and TLR7 ligands	s.c.	OVA + ADJ	↑CD8, ↓growth B16-OVA or B16.F10 (T)	[198]
*Chemokine related* Fusion of chemokine MCP3 or IP10 to lymphoma-derived scFv as protein or DNA plasmid	s.c. or i.d.	scFV	↑humoral, ↓growth 38C-13 and A20 (P)	[199]
OVA with mAb or chemokine ligand XCL1 against XCR1	i.v.	OVA + ADJ	↑CD8, ↓growth E.G7 (P)	[200]

P: prophylactic, T: therapeutic, fp: footpad, i.v.: intravenous, s.c.: subcutaneous, i.p.: intraperitoneal, i.m.: intramuscular, EP: electroporated, i.d.: intradermal, i.n.: intranodal, i.t.: intratumoral, U: unknown, ADJ: adjuvant, CPM: cyclophosphamide iNKT: induced natural killer T cell, and in bold: all studies with human APCs.
